# Macroscopic, Histologic, and Immunomodulatory Response of Limb Wounds Following Intravenous Allogeneic Cord Blood-Derived Multipotent Mesenchymal Stromal Cell Therapy in Horses

**DOI:** 10.3390/cells10112972

**Published:** 2021-11-01

**Authors:** Suzanne J. K. Mund, Daniel J. MacPhee, John Campbell, Ali Honaramooz, Bruce Wobeser, Spencer M. Barber

**Affiliations:** 1 Department of Large Animal Clinical Sciences, Western College of Veterinary Medicine, University of Saskatchewan, Saskatoon, SK S7N 5B4, Canada; john.campbell@usask.ca (J.C.); spence.barber@usask.ca (S.M.B.); 2 Department of Veterinary Biomedical Sciences, Western College of Veterinary Medicine, University of Saskatchewan, Saskatoon, SK S7N 5B4, Canada; d.macphee@usask.ca (D.J.M.); ali.honaramooz@usask.ca (A.H.); 3 Department of Veterinary Pathology, Western College of Veterinary Medicine, University of Saskatchewan, Saskatoon, SK S7N 5B4, Canada; bruce.wobeser@usask.ca

**Keywords:** stem cells, cytokines, wound healing, fibrosis, exuberant granulation tissue, equine, MSCs

## Abstract

Limb wounds are common in horses and often develop complications. Intravenous multipotent mesenchymal stromal cell (MSC) therapy is promising but has risks associated with intravenous administration and unknown potential to improve cutaneous wound healing. The objectives were to determine the clinical safety of administering large numbers of allogeneic cord blood-derived MSCs intravenously, and if therapy causes clinically adverse reactions, accelerates wound closure, improves histologic healing, and alters mRNA expression of common wound cytokines. Wounds were created on the metacarpus of 12 horses. Treatment horses were administered 1.51–2.46 × 10^8^ cells suspended in 50% HypoThermosol FRS, and control horses were administered 50% HypoThermosol FRS alone. Epithelialization, contraction, and wound closure rates were determined using planimetric analysis. Wounds were biopsied and evaluated for histologic healing characteristics and cytokine mRNA expression. Days until wound closure was also determined. The results indicate that 3/6 of treatment horses and 1/6 of control horses experienced minor transient reactions. Treatment did not accelerate wound closure or improve histologic healing. Treatment decreased wound size and decreased all measured cytokines except transforming growth factor-β3. MSC intravenous therapy has the potential to decrease limb wound size; however, further work is needed to understand the clinical relevance of adverse reactions.

## 1. Introduction

Traumatic cutaneous limb wounds occur frequently in horses and often develop complications [1]. Compared with wounds on the body, limb wounds have a prolonged lag phase [2,3,4] where the wound edges retract and the wound initially becomes larger and then decreases back to the original size. The wound then continues to heal through further contraction and then epithelialization [5,6]. A cutaneous wound healed primarily with epithelialization has a larger scar and is more fragile and weaker than unwounded skin [7] and has a poorer cosmetic and functional outcome, as it consists mainly of acellular collagen and is devoid of hair follicles and sebum and apocrine glands [5]. Limb wounds also are more likely to develop exuberant granulation tissue, believed to be secondary to prolonged low-grade inflammation and abnormal angiogenesis [1,8], leading to hypoxia and upregulation of profibrotic cytokines [4,9].

Multipotent mesenchymal stromal cells (MSCs) have recently been investigated as ancillary treatment to improve wound healing in horses [10,11]. Horses are fractious by nature, and performing procedures on the limbs carries a significant risk of injury to the clinician. Intravenous (IV) jugular administration is technically simple and has been shown to accelerate and improve cutaneous healing in laboratory animal wound models [12,13] through local and systemic immunomodulation [14,15,16]. Allogeneic cord blood-derived MSCs (allo-CB-MSCs) are promising for clinical use, as they can be collected noninvasively, be characterized, expanded, and stored long-term, and be administered shortly after an injury during the acute inflammatory phase, when MSC therapy may be most effective [17,18,19]. In the equine literature, several studies have been performed to determine the recipients’ responses to IV MSC administration [20,21,22,23], but none have determined whether IV MSC therapy accelerates cutaneous wound healing or influences the local immune response.

Limb wounds in horses share inflammatory characteristics with chronic wounds in humans; specifically, a low-grade protracted inflammatory response increases infection risk and impairs epithelialization, angiogenesis, and contraction [1,15,24,25]. In an acute equine limb wound, the initial inflammatory response is less robust than a body wound, and it is believed that this lesser response is inadequate to trigger the cytokine cascade required to resolve the inflammatory phase and transition into each of the sequential phases of healing, leading to a chronic nonhealing wound [1,25]. MSC therapy in laboratory animals has been shown to transition chronic wounds from a proinflammatory and proliferative cytokine environment to one that resolves inflammation and promotes remodeling [16,24].

The effective cell dose of IV MSCs required to influence wound healing in horses is unknown. Currently, the highest reported dose administered IV to an equid is 1.02 × 10^8^ cells per animal [26], with no clinical adverse responses. Furthermore, the cytokine profile of healing limb wounds in horses has not been reported. The primary objectives of this study were to determine whether 1.51–2.46 × 10^8^ allo-CB-MSCs administered IV following experimental limb wound creation would (1) cause a clinically detectable adverse response, (2) influence epithelialization and contraction rates and hence wound closure, and (3) influence histologic healing characteristics. A secondary objective was to record gene expression levels of proinflammatory (chemokine C-X-C motif ligand (CXCL)8, tumor necrosis factor (TNF)-α), anti-inflammatory (interleukin (IL)-4, IL-10), inflammation resolving (CXCL10, interferon (IFN)-γ), and fibrosis-related (profibrotic-transforming growth factor (TGF)-β1, TGF-β2; antifibrotic TGF-β3) cytokines over time and determine whether IV allo-CB-MSC therapy would significantly alter their expression pattern. Our hypotheses were that IV allo-CB-MSC therapy would not cause a clinically adverse response, would accelerate wound closure, and would improve histologic healing characteristics.

## 2. Materials and Methods

### 2.1. Recipient Animals

**Figure 1:**
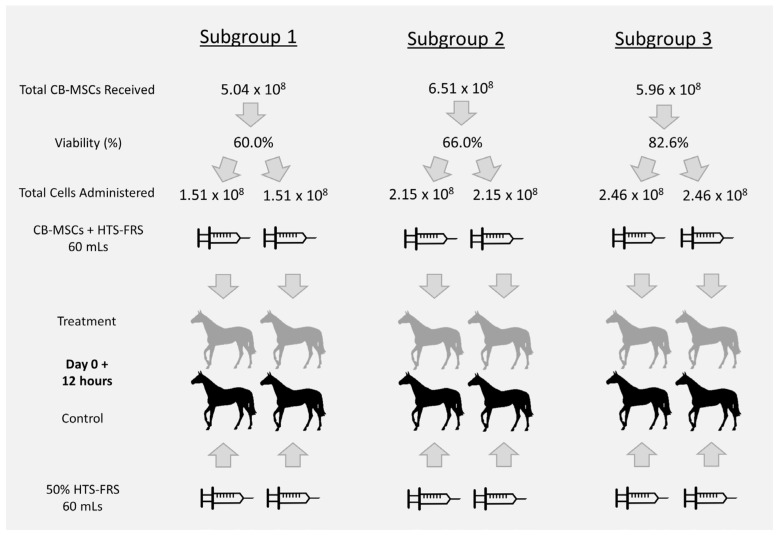
Basic schematic of division of horses into treatment and control subgroups and total number of allo-CB-MSCs administered. Twelve horses were randomly placed into a treatment and control group and then further divided into three subgroups. Following shipment of cells, a total cell count and assessment for viability were performed. The total cells were evenly divided, suspended in 50% diluted HypoThermosol FRS (HTS-FRS), and administered IV to the treatment horses. The control horses were administered a placebo IV injection of 50% diluted HTS-FRS.

A total of 12 female adult Quarter Horses (3–16 years; mean 8.3 +/− 3.5 SD) with no physical evidence of past injury to their limbs were acquired. Horses were healthy on physical exam and had normal complete blood counts and serum chemistries at the time of surgery. Horses were randomized (www.random.org/sequences; accessed on 23 June 2016) into even treatment and control groups. To accommodate constraints of facility housing and anesthesia personnel, and to allow time for expansion and shipment of sufficient numbers of allo-CB-MSCs, horses were further divided into three subgroups consisting of two treatment and two control horses (subgroups 1, 2, and 3) (Figure 1).

Then, 12 h prior to surgery, a 14-gauge IV catheter was placed aseptically in the jugular vein and feed withheld for 8 h prior to induction of anesthesia. As the created wounds were objectively small and discomfort relatively minor, the wounds were bandaged only for recovery then left unbandaged to not influence exuberant granulation tissue formation [1,27]. Similarly, no analgesics were administered to not affect inflammation associated with healing [28,29].

### 2.2. Wound Creation

**Figure 2:**
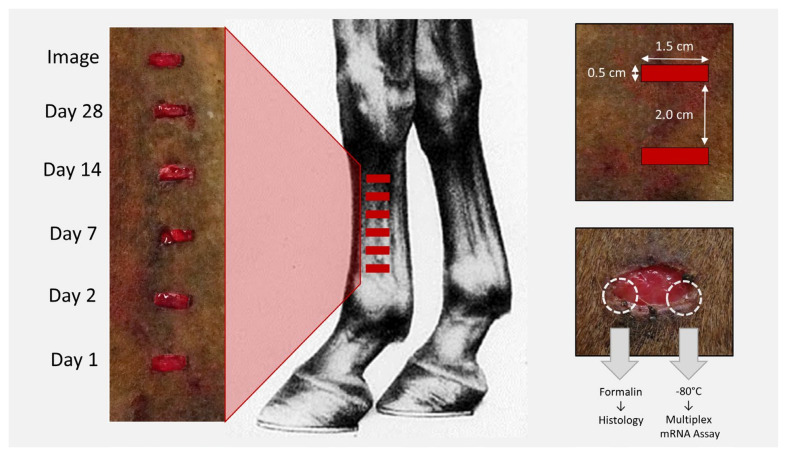
Basic schematic of wound creation and sequence of biopsy collection. On day 0, six wounds were created on the left forelimb of each horse measuring 0.5 cm × 1.5 cm and placed 2 cm apart in a vertical orientation. On days 1, 2, 7, 14, and 28, a biopsy was collected from both the dorsal and palmar aspects of the wound in a distal to proximal sequence. The dorsal biopsy was submitted for histology and the palmar biopsy was snap frozen for later cytokine mRNA multiplex assays. The top wound was left to heal by second intention and imaged on days 7, 14, and 28 and assessed by planimetric analysis for rate of wound closure.

On day 0, horses were anaesthetized routinely (sedated with 1.1 mg/kg xylazine IV, followed by 2.2 mg/kg ketamine + diazepam 0.02 mg/kg IV) and maintained on total IV anesthesia (1 L 5% guaifenesin + 1000 mg ketamine + 500 mg xylazine at 1.4–2.8 mL/kg/h to effect). After clipping and prepping the area aseptically, six full-thickness excisional skin wounds were created on the lateral aspect of the left metacarpus measuring 0.5 × 1.5 cm in a horizontal orientation in a vertically stacked arrangement 2.0 cm apart. A portion of excised skin was divided, and one segment was placed in formalin for histologic evaluation, and the other snap frozen and stored at −80 °C for later cytokine multiplex messenger RNA (mRNA) assays of baseline aforementioned cytokines (Figure 2). Similarly, wounds were created on the left lateral thorax at the site of the 10th costochondral junction for purposes of studying thoracic wound healing characteristics for a separate study, the results of which are not reported in this manuscript.

### 2.3. Source of Allo-CB-MSCs, Administration, and Monitoring

Allogeneic CB-MSCs originating from five unrelated male donor foals (eQcell Inc.; Guelph, ON, Canada) were characterized, prepared, processed, and transported overnight in full concentration HypoThermosol FRS (HTS-FRS) (BioLife Solutions, Bothell, WA, USA) in a cooler approved for transport of mammalian cells (Greenbox 2–8 °C thermal management system, ThermoSafe, Arlington Heights, IL, USA) as previously described [26,30,31]. Briefly, CB-MSCs were expanded from cryopreserved sources, and after culture showed a consistent phenotype, had trilineage differentiation capabilities, and had high expression of cluster of differentiation (CD)20, CD44, CD90, and low expression of CD4, CD8, CD11, CD73, major histocompatibility complex (MHC)-I and MHC-II [30,31]. Cells were expanded in Dulbecco’s modified eagle medium containing 30% fetal bovine serum, 1% penicillin, 1% streptomycin, and 1% L-glutamine [30,31] until approximately 6.00 × 10^8^ CB-MSCs were achieved. Cells were passaged 4–5 times and cultured for 35–45 days. CB-MSCs were then harvested using trypsin and ethylenediaminetetraacetic acid, washed with phosphate-buffered saline, and pooled into a mixed population of donor cells [21,26,31]. Cells were then resuspended in HTS-FRS prior to being placed in the cooler and shipped to our laboratory. Upon arrival, a small sample was collected, and viability was determined using a trypan blue exclusion assay and a hemocytometer counting chamber. Cells were then stored at 4 °C in a temperature-controlled laboratory refrigerator for approximately 8 h until injection 12 h after wound creation. Previous studies had determined that storing MSCs in HTS-FRS at 4 °C for up to three days had minimal effect on viability [21,32,33,34,35]. Immediately prior to injection, cells were resuspended without filtering to prevent cell loss in 50% diluted HTS-FRS chilled at 4 °C, to a total volume of 60 mL, and immediately placed on ice for transportation to stall side. Suspended allo-CB-MSCs were administered to treatment horses, and control horses were administered a placebo injection of 60 mL 50% diluted HTS-FRS, over 15 min (4 mL/min) via the indwelling catheter. Vital parameters were measured prior to injection and then every minute for the first five minutes, followed by every five minutes until the administration of the suspension was complete. If there were signs of an adverse reaction (tachycardia, tachypnea, hyperthermia, colic, urticaria, fasciculations, agitation/anxiety), the administration was suspended until symptoms abated, then continued at a slower rate until complete. A physical exam was performed every 12 h for the first 7 days, followed by a distance exam daily until the conclusion of the study on day 28. Total cells received and administered to each subgroup are reported in Figure 1.

### 2.4. Wound Closure Analysis

**Figure 3:**
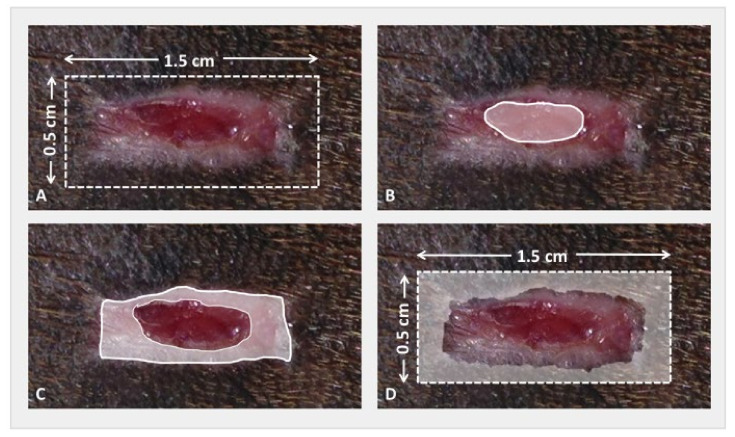
Representative diagram of measured areas of wound to determine proportion of wound closure by contraction and epithelialization using calibrated planimetric analysis: (**A**) representative area of the originally created wound (day 0) in relation to the current (day x) measured wound; (**B**) shaded area represents area of open granulation tissue; (**C**) epithelialization was determined by measuring the total area of nonhaired wound area and then subtracting open granulation tissue previously measured in (**B**,**D**) total contraction was determined by subtracting both the previously measured open granulation tissue in B and epithelialized tissue in C from the originally created wound size in A (day 0).

Photographs were taken of the proximal-most wound, which was never biopsied, with a metric ruler adjacent to the wound for wound size calibration on days 7, 14, 21, and 28. Days to complete wound closure were recorded when epithelium from the borders of the wound covered the granulation tissue bed and met at the center of the wound. Images were randomized, and the wound areas were measured in triplicate by a blinded evaluator. Using planimetric analysis (Image-Pro^®^, Media Cybernetics, Inc., Rockville, MD, USA), the percentage of closure by contraction and epithelialization was determined (Figure 3). The following formulas were used:

Percent Epithelialization
  (Area of the nonhaired epithelialized wound on day (x) mm^2^ − Area of open granulation tissue on day (x) mm^2^)/Area of the originally created wound on day 0 mm^2^ × 100.
Percent Contraction
  Area of the originally created wound on day 0 mm^2^ − Area of the nonhaired epithelialized wound on day (x) mm^2^/Area of the originally created wound on day 0 mm^2^ × 100.

### 2.5. Biopsy Collection

Biopsies were collected during the per acute (day 1) and acute (day 2) inflammatory phase, during the mid- (day 7) and late (day 14) proliferative phase, and the early (day 28) remodeling phase. At each time point, treatment and control horses were sedated (0.01 mg/kg detomidine + 0.01 mg/kg butorphanol IV), and biopsies were collected from one site in a distal to proximal sequence after subcutaneous infiltration of 2% lidocaine (Figure 2). Wounds were biopsied sequentially rather than randomly in order to expose all biopsied wounds to identical local inflammation from previously biopsied sites and to avoid dependent edema. Two biopsies were collected from each site—one from the dorsal and one from the palmar margin using a 6 mm punch biopsy instrument that included approximately half skin and half wounded tissue. The dorsal biopsy was preserved in formalin for histopathology and the palmar portion snap frozen and stored at −80 °C for cytokine multiplex mRNA assays (Figure 2).

### 2.6. Histologic Analysis

Biopsies were randomized by treatment and time then graded by a blinded board-certified veterinary pathologist. A grading scale modeled after other wound grading scales (Table 1) [11,36] was created based on the progression of repair and inflammation. More specifically, the total repair score was the sum of epithelialization, collagen, and vascularization scores, and a total inflammation score was the sum of vascular and cellular inflammation scores.

### 2.7. Cytokine Multiplex mRNA Assays

Messenger RNA of proinflammatory, anti-inflammatory, inflammation resolving, profibrotic, and antifibrotic cytokines were isolated from the frozen biopsies using an assay processing kit according to the manufacturer’s protocol (QuantiGene Plex; Thermo Fisher Scientific, Santa Clara, CA, USA) for each of the biopsy collection days. Assays were performed in duplicate using equine-specific primers. Samples were analyzed (Bioluminex; Bio-Rad Laboratories Ltd., Mississauga, ON, Canada) and results normalized to hypoxanthine–guanine phosphoribosyltransferase and expressed as combined mean fold change (MFC) for each cytokine category.

### 2.8. Statistical Analysis

Data were entered into a commercial spreadsheet (Microsoft Excel, Version 2016; Microsoft Corporation, Redmond, WA, USA). Statistical analysis was performed with a commercial software package (Stata 15.1; StataCorp, College Station, TX, USA). Data from treatment and control subgroups were combined into a single treatment group and single control group, as there was minimal subgroup variation. The outcome variable of days to complete wound closure was normally distributed (Shapiro–Wilk test), and treatment and control groups were compared (two-sample *t*-test; significance *p* ≤ 0.05). Histologic healing and inflammation scores for each day were evaluated (Wilcoxon rank sum; significance *p* ≤ 0.05). The MFC of cytokines, area of the nonhaired portion of the wounds, and percentage of contraction and epithelialization were analyzed with a generalized estimating equation (GEE) model (Gaussian data distribution, exchangeable correlation structure, and robust standard errors). Variables for consideration in the GEE model were the treatment group (treatment vs. control) and the day of measurement. Interaction effects for treatment group and day were evaluated and in cases in which significant interaction existed, an overall treatment effect was not reported. To avoid overinterpretation, a liberal limit of *p* ≤ 0.2 for the overall treatment effect of data combined from all measured days was set as the threshold to further consider the effect of treatment at each individual day within the GEE model. Pairwise comparison (*p* ≤ 0.05) was performed for individual days if an overall treatment effect was determined to be present. Outcomes from GEE are reported as linear predicted contrast (95% CI) between the treatment and control groups. Differences in measures were estimated with the contrast of predicted margins.

## 3. Results

### 3.1. Allo-CB-MSC Administration and Monitoring

All horses remained normothermic during administration. Three horses (3/6) in the treatment group (two in subgroup 2 and one in subgroup 3) and one horse (1/6) in the control group (subgroup 3) experienced transient infusion reactions (Table 2). In all instances, the administration was suspended until symptoms abated (5–15 min) and then continued at a slower rate. All horses returned to normal vital parameters shortly after completion of administration and had normal physical exams for the remainder of the study.

### 3.2. Wound Closure Analysis

One of the treatment horses in subgroup 3 sustained a large degloving injury on the lateral aspect of the right neck on day 22 requiring nonsteroidal anti-inflammatory therapy and basic wound care. As systemic inflammation created by the neck wound, and treatment with anti-inflammatories may have influenced healing of the experimental limb wounds and confound results, the data collected from her were subsequently eliminated from analysis for the remainder of the study.

**Figure 4:**
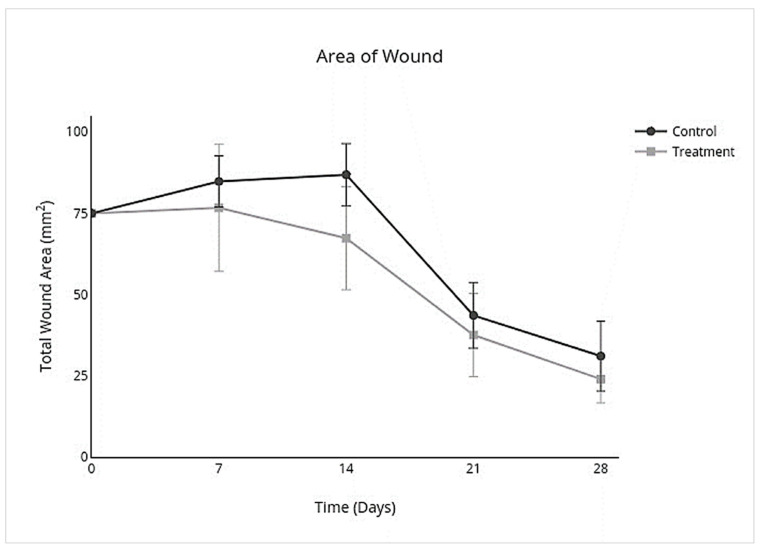
Total area (mm^2^) of limb wounds. There was an overall decrease in total nonhaired wound area over all days but no treatment effect on individual days. Both treatment and control wounds had an increase in nonhaired wound area during the lag phase but were less pronounced in the treatment group.

**Figure 5:**
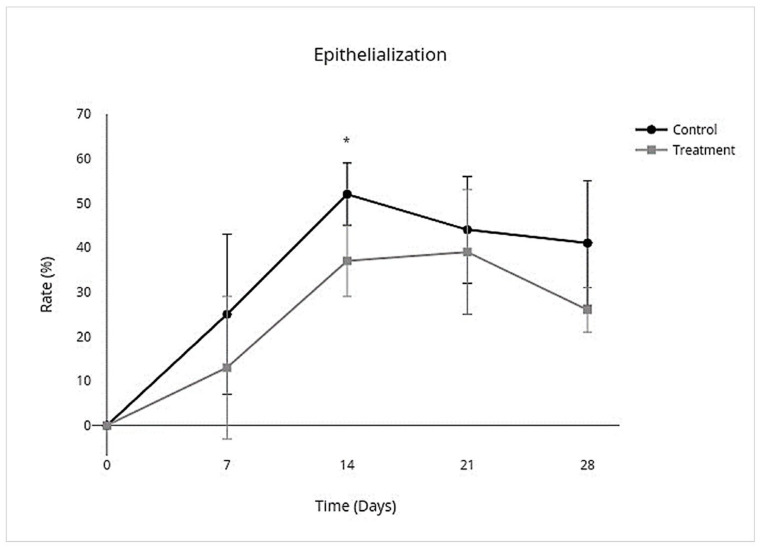
Total area (%) of epithelialization contributing to wound closure of limb wounds. There was an overall decrease in total epithelialization over all days with individual treatment effect on day 14 (*p* = 0.02). (*) = *p* < 0.05.

**Figure 6:**
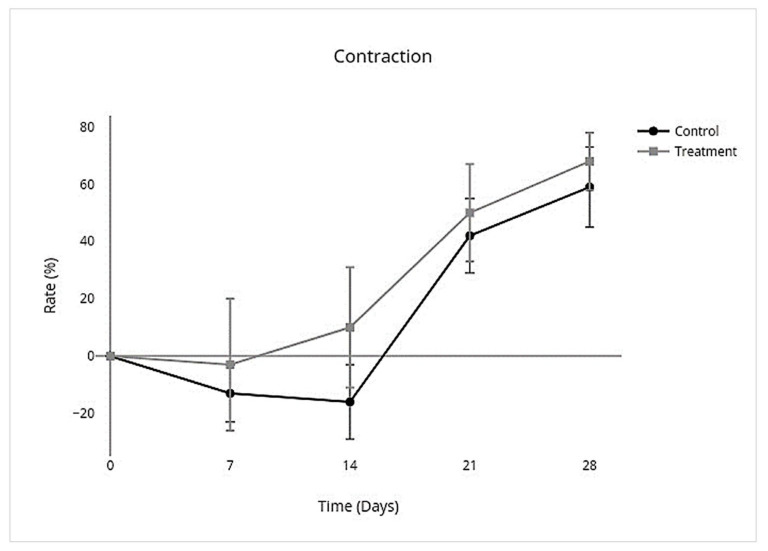
Total area (%) of contraction contributing to wound closure of limb wounds. There was an overall increase in total contraction over all days with no significant treatment effect on individual days.

In the control group, wounds had complete closure on day 27 (mean 27; SD ± 3), and the treatment group had complete closure on day 26 (mean 26; SD ± 4). There was no significant difference between treatment and control groups for the number of days to complete closure (95% CI, −4.1 to 5.8; *p* = 0.7). The nonhaired wound size was larger than the originally created wound size in both the treatment and control groups on day 7 and continued to become larger in the control group by day 14 but became smaller than the original wound size in the treatment group by day 14 (Figure 4). An overall treatment effect over all days was seen for nonhaired wound size (95% CI, −24.0 to 3.5; *p* = 0.1), but there was no interaction on individual days (Table 3; Figure 4). Allo-CB-MSC therapy had an overall treatment effect for less epithelialization over all days (95% CI, −0.21 to −0.023; *p* = 0.02) with a significant treatment effect on day 14 (95% CI −0.28 to −0.019; *p* = 0.02) (Table 3; Figure 5), and an overall treatment effect for more contraction over all days (95% CI, −0.046 to 0.31; *p* = 0.1) was characterized by a less pronounced lag phase in the treatment group than the control group, although no significant interaction was identified on individual days (Table 3; Figure 6).

### 3.3. Histologic Analysis

Histologic repair and inflammation scores between each group were nearly identical at each of the days; thus, statistical analysis was invalid, as data could not be ranked. Descriptive data are only presented (Table 4). In both treatment and control groups, the repair score increased, and the inflammation score decreased throughout time.

### 3.4. Cytokine Multiplex mRNA Assays

**Figure 7:**
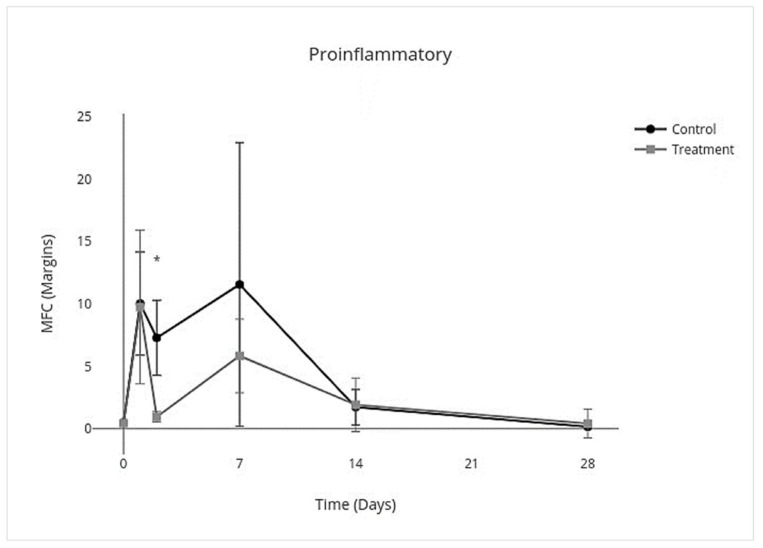
MFC of mRNA expression of proinflammatory cytokines over time following IV allo-CB-MSC therapy. In the treatment group, there was an overall treatment effect over all days, characterized by decreased expression of proinflammatory cytokines with an individual significant treatment effect on day 2 (*p* = 0.03). (*) = *p* < 0.05.

**Figure 8:**
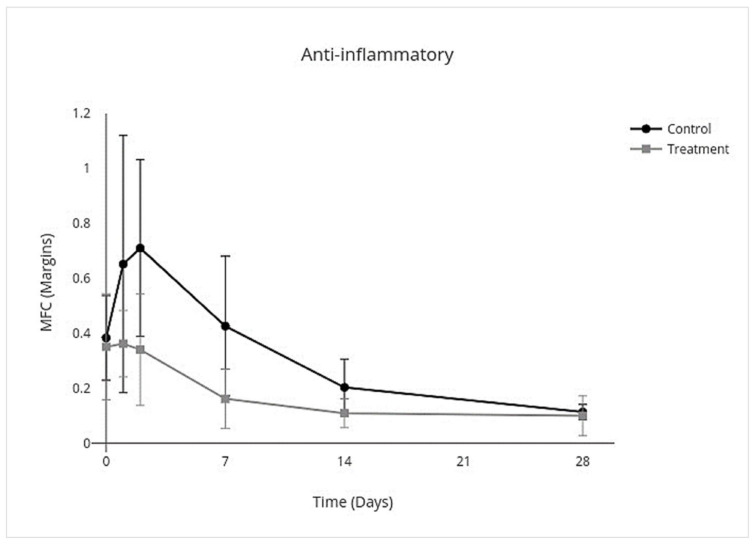
MFC of mRNA expression of anti-inflammatory cytokines over time following IV allo-CB-MSC therapy. In the treatment group, there was an overall treatment effect over all days, characterized by decreased expression of anti-inflammatory cytokines with no significant treatment effect on individual days.

**Figure 9:**
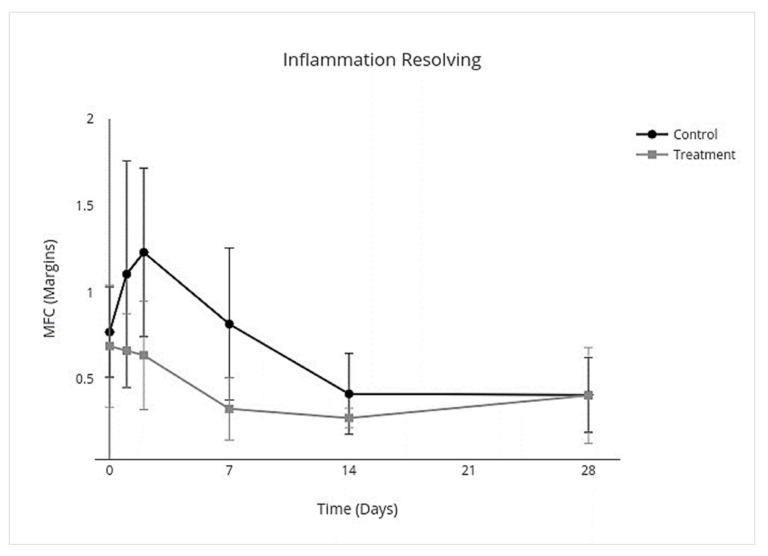
MFC of mRNA expression of inflammation resolving cytokines over time following IV allo-CB-MSC therapy. In the treatment group, there was an overall treatment effect over all days, characterized by decreased expression of inflammation resolving cytokines with no significant treatment effect on individual days.

**Figure 10:**
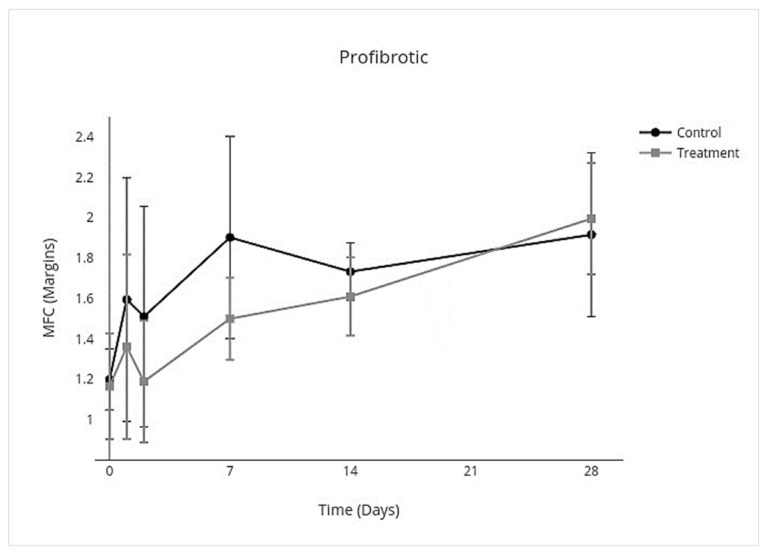
MFC of mRNA expression of profibrotic cytokines over time following IV allo-CB-MSC therapy. In the treatment group, there was an overall treatment effect over all days, characterized by decreased expression of profibrotic cytokines with no significant treatment effect on individual days.

**Figure 11:**
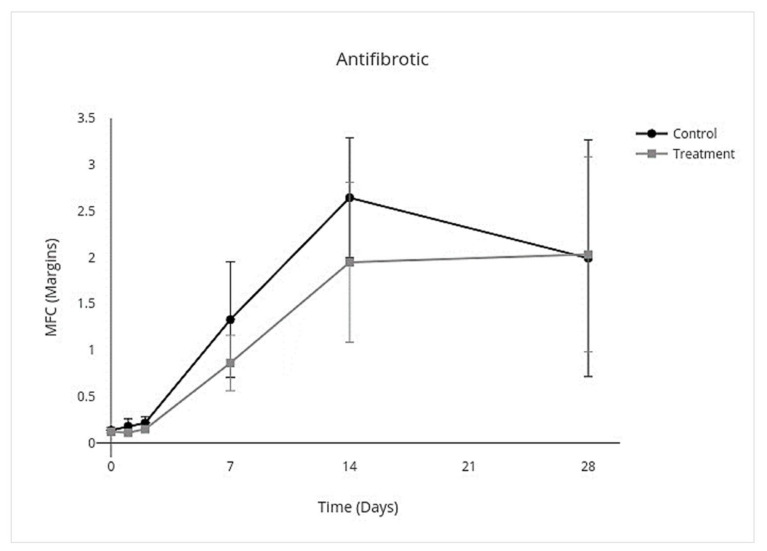
MFC of mRNA expression of antifibrotic cytokines over time following IV allo-CB-MSC therapy. In the treatment group, there was neither overall treatment effect over all days nor significant treatment effect on individual days.

IV allo-CB-MSC administration had an overall treatment effect over all days on proinflammatory cytokines where proinflammatory mediators were lower (95% CI, −3.8 to −0.17; *p* = 0.03) with an individual treatment effect on day 2 (95% CI, −10.0 to −2.3; *p* < 0.001) (Figure 7). An overall treatment effect over all days was measured where anti-inflammatory mediators were also lower (95% CI, −0.33 to −0.025; *p* = 0.02), but there was no treatment effect on individual days (Figure 8). An overall treatment effect over all days was measured where inflammation-resolving mediators were lower (95% CI, −0.56 to −0.023; *p* = 0.03), but again there was no treatment effect on individual days (Figure 9). Similarly, an overall treatment effect over all days was measured where profibrotic mediators were lower (95% CI, −0.43 to 0.086; *p* = 0.2), but there was no interaction on individual days (Figure 10). There was no treatment effect over all days for antifibrotic mediators, so treatment effect on individual days was not further investigated in the GEE model (Figure 11; Table 5).

## 4. Discussion

Contrary to our hypothesis, following IV administration of 1.51–2.46 × 10^8^ allo-CB-MSCs, 3/6 of the treatment horses and 1/6 of the control horses experienced mild transient adverse reactions, and healing was not accelerated or improved histologically. However, over all days, treatment horses had improved contraction, allowing for less epithelialization, which resulted in smaller immature scars, compared with controls. Additionally, over all days, treatment horses had lower proinflammatory, anti-inflammatory, inflammation-resolving, and profibrotic cytokines. To the best of the authors’ knowledge, this is the highest dose via any route experimentally administered to horses. Furthermore, this is the first study to demonstrate an immunomodulatory response and decreased immature scar size of equine limb wounds following IV allo-CB-MSC therapy.

Allogeneic MSCs were chosen for this study rather than autologous MSCs because allo-MSCs can be administered during the acute inflammatory stage when MSC therapy may have its most beneficial effects [17,19,37,38]. Although allo-MSCs are MHC-I and MHC-II negative in vitro [39], MHC expression upregulates after engraftment and interacts with endogenous mononuclear cells, leading to early rejection and immune response [21,39,40,41,42]. Despite this finding, allo-MSC therapy often has favorable outcomes when compared with controls [41,43], and has had promising outcomes in equine experimental synovitis [44,45] and naturally occurring arthritis studies [37,46] by altering the inflammatory environment. Although repeat administrations of allo-MSCs may induce MHC-induced acquired cell-mediated and humoral immune responses [47], a single dose of allo-MSCs at the time of injury would allow time for collection, culture, and expansion of autologous MSCs for later repeat dosing.

The cell dose in this study was determined by the maximum amount of CB-MSCs that the supplier could provide as resources and facilities allowed. In studies with laboratory mice, typically 1 × 10^6^ cells are administered per mouse [16], equivalent to 33 × 10^6^ cells per kilogram. Administering the same dose to a horse would be cost prohibitive and technically challenging, but we believe a clinically measurable response is more likely to be measured with an IV dose higher than what has currently been administered to horses, provided there are no significant adverse reactions.

Interestingly, one of the six control horses had an adverse clinical response to the placebo (Table 2). Other studies examining IV MSC administration in horses did not report any adverse reactions, although the doses administered were relatively small [20,21,22,23,26]. Regardless, within the treatment group, potential adverse reactions were anticipated secondary to MHC reaction [39,42] and/or pulmonary vascular injury [48]. Clinical response was not anticipated in the control group, as the placebo contained only chilled diluted HTS-FRS, and reactions were not reported in two other studies where horses received IV MSCs suspended in HTS-FRS [21,26]. A thermic response to a chilled IV solution may have caused the responses in both groups, although unlikely as saline chilled to 0 °C is commonly administered to critically ill human patients to measure the cardiac output with rare complications [49,50]. Another possible cause is a physiologic response to concentrated electrolytes within the perfusate. The composition of HTS-FRS is not publicly available but is similar to the original HypoThermosol [21,51]. The composition of the original HypoThermosol had a concentration of 42.5 mEq/L potassium [51]. The safe dose of potassium is 0.5 mEq/kg/h [52], or 3.75 mEq/min for the average 450 kg horse. Administration of 60 mLs of 50% diluted HTS-FRS over 15 min would be equivalent to 0.085 mEq/min for the average-sized horse, well within the safe rate. In our study, live cells were not filtered from the delivered solution to avoid further cell stress and cell death. Lysed cells release a significant amount of intracellular potassium that may have caused transient symptoms of hyperkalemia in the treatment group but would not have altered the cell-free HTS-FRS administered to the control group. It is also possible that the HTS-FRS contains a preservative that causes an immunological response in some individuals. Additionally, interestingly, the absence of clinical response in the first subgroup may be related to the lower total dose of viable CB-MSCs, and hence reduced expression of MHC-II and/or fewer intact cells entering the pulmonary vasculature. Regardless of the cause, the adverse reactions experienced were transient and mild, although one should consider administering the suspension at a slower rate to potentially avoid these reactions.

**Figure 12:**
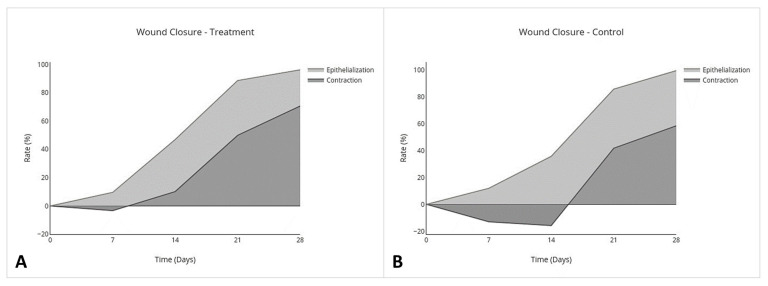
Total area (%) of contraction and epithelialization contributing to wound closure of treatment and control limb wounds. There was an overall increase in total contraction and epithelialization over all days in the treatment group (**A**). The lag phase was less pronounced in the treatment group, and wound closure was healed with approximately 75% contraction and 25% epithelialization (**A**), compared with 60% and 40% of contraction and epithelialization, respectively, in the control group (**B**).

Limb wound closure in the treatment group was characterized by a less pronounced lag-phase in the first two weeks, and by day 28, the treatment group had healed with approximately 75% contraction and 25% epithelialization (Figure 12B), whereas the control group had approximately 60% contraction and 40% epithelialization (Figure 12A). Determining the direct relationship between cytokine expression and wound closure was beyond the scope of this project, but there are some interesting differences in the cytokine profiles of equine limb wounds following IV MSC infusion compared with studies using other animal models. To the authors’ knowledge, no comparable wound cytokine analysis studies have been performed in horses following IV MSC therapy. In laboratory animal wound models that received MSC therapy acutely, cytokine profiles typically had decreased proinflammatory mediators but increased anti-inflammatory and profibrotic mediators [13,15,16,24], in contrast to the decreased anti-inflammatory and profibrotic cytokines that we measured. This may be in part due to the “first-pass effect”, in which following IV infusion, MSCs become entrapped in the lungs, but still suppress inflammation through secretion of anti-inflammatory cytokines, which are absorbed by the pulmonary vasculature and distributed systemically, having effects at local injury sites [16,19]. MSCs also modify the immune response through “licensing”, where activated MSCs secrete anti-inflammatory cytokines after exposure to proinflammatory cytokines [53]. This process is somewhat dose dependent on exposure to TNF-α and/or IFN-γ. Although speculative, it is possible that our treated horses had a lessened systemic proinflammatory response after MSC lung entrapment, which then likewise decreased a reciprocated licensed release of endogenous anti-inflammatory and inflammation-resolving cytokines at the wound site. This cytokine profile may have promoted the trends for increased contraction in the treatment group. Further studies examining the relationship between cytokine profile and wound closure are needed.

Allogeneic CB-MSC therapy did not affect TGF-β3 expression. TGF-β3 promotes the maturation of granulation tissue and minimizes scar formation, and is expressed by M2 macrophages that are involved in resolving inflammation and promoting fibrosis during the later stages of wound healing [5,54]. In this study, cells were administered during the acute phase of wound healing when M2 macrophages were not present. IV MSC therapy during later stages of wound healing or to treat chronic nonhealing wounds may have an appreciable effect on TGF-β3 expression that we did not detect in this study model.

Following treatment, expression of inflammatory mediators was lower over all days for all inflammatory mediators except TGF-β3, and positive effects on immature dermal scar size were still appreciated—specifically, increased contraction and decreased epithelialization. This finding challenges the theory that limb wounds need more acute inflammation to promote healing [1,2]. We are currently researching the local cytokine environment of limb wounds compared with thoracic wounds, with and without IV allo-CB-MSC treatment, to further understand the role of MSCs and cytokine profiles in wound healing, and will be described in future articles.

IV jugular administration was chosen for this study for its ease of administration and decreased risk to the administrator; horses are fractious by nature and can injure the clinician performing procedures on the limb even if the horse is adequately sedated and restrained. Furthermore, placing an intraarterial catheter in order to avoid the first-pass-effect to the lungs is a very challenging procedure in horses, which typically requires a general anesthetic and often has complications such as hematoma formation, thrombosis, and vasospasm [55,56,57]. However, if MSC therapy is more effective using alternate localized methods, such as direct intralesional injection and regional limb perfusion (RLP), the clinician may choose to perform them despite the risk. Studies examining alternate methods for treating limb wounds with MSCs are lacking in the equine literature, but we can extrapolate from tendon injury models to predict possible outcomes. Compared with RLP and direct intralesional injection, IV delivery appears to have the lowest rates of engraftment [16,58]. Using a naturally occurring tendinopathy clinical trial, Becerra et al. tracked technicium99m-labeled MSCs using nuclear scintigraphy following IV jugular administration, RLP, and direct intralesional injection to the tendon lesions. They found that there was no homing following IV administration, small amounts following RLP, and the most after direct intralesional injection [58]. However, the horses were only administered 1.0 × 10^7^ MSCs, and the injuries were not considered acute [58], both factors that would negatively influence homing and engraftment [16,59,60]. Homing and persistence of MSCs seem to be most efficient with direct intralesional injection in tendons in horses [56,58,61,62]. However, a direct intralesional injection can be used only if the lesion is discrete and accessible by needle, and there is concern that injecting a substance into already compromised tissue may cause further iatrogenic injury [16,57,63]. It is fair to assume that introducing a substance into compromised wounded cutaneous tissue may also have a negative effect on the vital angiogenesis that is occurring during the acute phases. RLP can successfully deliver MSCs to an active lesion, and although homing and engraftment appears to be less efficient than direct injection [56,58], RLP may be more advantageous in cases where the lesion cannot be easily accessed with a needle, such as in the hoof capsule, or where there is diffuse disease or injury while decreasing the risk of an adverse response from large doses of jugular MSCs. Furthermore, fewer cells may be required to have the same effect as jugular IV administration, although the systemic benefits may not be appreciated [16]. Although successful homing and engraftment of MSCs is possible following IV jugular administration, exploring other delivery methods may improve wound healing while avoiding possible systemic adverse reactions.

Preconditioning CB-MSCs prior to injection may improve engraftment and hence wound healing outcomes. The purpose of preconditioning is to either increase the cells’ efficiency in homing and transendothelial migration and/or improve the resilience to rejection in the host tissue by exposing the cells to certain conditions and substances [53,59,60]. Exposing the cells to hypoxic conditions prior to injection preconditions them to tolerate a hypoxic environment and causes upregulation of CXCR4, a receptor necessary for transendothelial migration [59,60]. In an equine wound model, however, hypoxic preconditioning did not enhance wound healing, although evidence of engraftment was not examined [11]. Other possible preconditioning or priming methods include exposure to substances including antioxidants, statins, and interferon, and gene therapy to prevent premature apoptosis and to upregulate surface receptors to enhance engraftment [53,59,60]. Methods to precondition the host tissue to be more receptive to foreign MSCs include irradiation, postischemia conditioning through repeated restriction of blood flow following MSC administration, and systemic statins [60]. Further studies examining the effects and feasibility of preconditioning of MSCs and the host tissue in horses with limb wounds are warranted.

In this study, a single dose of CB-MSCs was administered in the acute phase of wound healing, which has been shown to have more beneficial effects than dosing later [17,18,19,37,38,41]. However, several studies in other species have shown that repeat doses have even more beneficial effects than a single acute dose [19,64]. An equine wound model using repeat doses of MSCs during the acute inflammatory, inflammation resolving, and remodeling phases to determine whether increasing dosing regimens would further enhance healing and wound closure is indicated.

There were several challenges met in this study. First, the total amount of viable cells administered to treat horses in each subgroup varied from 1.51 × 10^8^ to 2.46 × 10^8^, a difference of 1.05 × 10^8^ cells. Previously, we had conducted feasibility studies, and a viability rate of 80% was expected following transportation [26]. However, on arrival, the first two shipments had 60% and 66% viability, far below the anticipated 82% viability rate in the third shipment. Other than an acknowledgment of the fragile nature of transporting large amounts of live mammalian cells, there was no immediate explanation for the variation. Admittedly, this may confound practicality for clinical practice. Regardless, the effective cell dose to accelerate healing time is unknown, and even the lowest administered dose in our horses was three times higher than the previously reported highest IV dose [21]. Second, we did not cross-match the administered cells to the recipient horses. It is possible three treatment horses failed to react because their haplotypes matched the MHC of the donor cells. However, this is extremely unlikely considering the donor cells were pooled from 5 separate donors and horses have upwards of 29 haplotypes [65], although it is possible that the severity of the reaction is related to whether there was an MHC mismatch of only one donor rather than all five. Third, mRNA expression was measured rather than translated functional proteins. However, recent studies have determined that in homeostatic mammalian cells, an mRNA to protein expression ratio can be predictably measured for different genes across all tissues, with 85% of a protein’s translation being determined at the mRNA level [66]. In addition to decreased technical complexity, improved measurement accuracy, and decreased cost of measuring mRNA using multiplex assay technology, compared with other methods such as qPCR [67,68], an 85% accuracy in predicting protein expression can be considered an acceptable screening tool before quantifying protein expression using more expensive and technically challenging methods such as ELISA. Fourth, although the scars were smaller in the treatment group by day 28, it would have been valuable to reexamine the scars when they had finished maturing in 6 to 12 months and determine if they remained smaller in the treatment group than the control group.

## 5. Conclusions

In conclusion, to the best of the authors’ knowledge, this is the first equine study to demonstrate changes in wound healing following IV MSC administration and also is the highest dose of MSCs (IV, allogeneic, or otherwise) ever administered to equids. We achieved our primary objective of administering 1.51–2.46 × 10^8^ allo-CB-MSCs IV, but contrary to our hypothesis, minor transitory adverse clinical reactions can occur, at least in our subpopulation of horses. The reasons for these adverse reactions are not clear, and because they were also seen in one control horse, the cause of the reactions may be related to the cell suspension solution, MHC mismatch, and/or hyperkalemia. Additionally, contrary to our hypothesis, the treated horses did not have accelerated wound closure or improved histologic healing, although they had smaller immature scar sizes, which may provide a cosmetically and functionally superior repair when matured. Furthermore, the cytokine profile was altered within these wounds to have less expression of all but antifibrotic mediators, which is in contrast to the current belief that more acute inflammation, followed by rapid resolution improves limb wound healing. Although a positive response was demonstrated, studies more closely examining the relationship between inflammation-associated cytokine expression must be performed before the usefulness and cost effectiveness of this therapy can be recommended as an ancillary treatment to cutaneous wound healing in horses. Further research investigating local methods of delivery is warranted, as they may be more cost effective and have improved outcomes, although the inherent risk to the clinician performing these procedures on the limbs of fractious horses needs to be carefully considered.

## Figures and Tables

**Table 1:** Histologic repair and inflammation grading scale.

Repair Grade						Inflammation Grade	
Score	Epithelialization	Score	Collagen	Score	Vascularization	Score	Inflammatory Response
0	No epithelium present	0	Absent	0	No activated endothelium	0	No inflammation present
1	<50% covering by epithelium	1	Minimal granulation tissue	1	Activated endothelium	1	Hemorrhage—no inflammatory cells
2	>50% covering by epithelium	2	Granulation tissue	2	Vascular proliferation with vertical orientation	2	Macrophage dominated
3	Bridging of excision	3	Fibroplasia with minimal collagen	3	Declining vascular proliferation	3	Admixed neutrophils/macro-phages
4	Keratinization	4	Return to normal dermal collagen	4	Return to normal vascular	4	Neutrophil dominated
Total = Repair Score						Total = Inflammation Score	

**Table 2:** Summary of adverse clinical responses following IV allo-CB-MSC administration.

Horse	Group	Subgroup	Symptoms Summary	Response to Decreased Infusion Rate
8	Treatment	2	Reaction following 28 mLs of infusion. Normothermic, mild tachycardia (48 bpm), moderate tachypnea (64 bpm), mild restlessness	Discontinued infusion for five minutes. Vital parameters returned to normal. Continued infusion. No alterations in vital parameters.
12	Treatment	2	Reaction following 20 mLs of infusion. Normothermic, mild tachycardia (48 bpm), moderate tachypnea (42 bpm), mild restlessness, mild colic symptoms (lip curling)	Continued infusion at a slower rate. Symptoms resolved within fifteen minutes.
7	Treatment	3	Reaction following 28 mLs of infusion. Normothermic, moderate tachycardia (60 bpm), moderate tachypnea (40 bpm), moderate restlessness, moderate colic symptoms (stretching, looking at flank)	Discontinued infusion for ten minutes. Heart rate and respiratory rate returned to normal. Continued infusion slowly. Colic symptoms resolved 15 min after infusion was completed.
11	Control	3	Reaction following 12 mLs of infusion. Normothermic, marked tachycardia (72 bpm), moderate tachypnea (40 bpm), marked restlessness, diffuse muscle fasciculations	Discontinued infusion for fifteen minutes. Muscle fasciculations resolved. Continued infusion slowly. Heart rate fluctuated between 48 and 60 bpm during infusion. Restlessness resolved shortly after complete infusion.

**Table 3:** GEE analysis of rate of limb wound closure by epithelialization and contraction following IV allo-CB-MSC administration.

	Non-Haired Wound Area (mm^2^)		Epithelialization (%)		Contraction (%)	
	95% CI	*p* Value	95% CI	*p* Value	95% CI	*p* Value
Overall Treatment effect	−24.0 to 3.5	0.1 *	−0.21 to −0.023	*p* = 0.02 *	−0.046 to 0.31	*p* = 0.1 *
Individual Day Treatment Effect	95% CI	*p* Value	95% CI	*p* Value	95% CI	*p* Value
Day 7	−35 to 19	0.9	−0.419 to 0.176	0.8	−0.22 to 0.42	0.9
Day 14	−43.0 to 4.0	0.1	−0.28 to −0.019	0.02 **	−0.054 to 0.57	0.1
Day 21	−27 to 15	0.9	−0.28 to 0.18	>0.9	−0.20 to 0.36	0.9
Day 28	−24.0 to 9.4	0.8	−0.34 to 0.041	0.2 *	−0.13 to 0.32	0.7

(*) = threshold for overall treatment effect over all days (*p* ≤ 0.2). (**) = indicates significant treatment effect on individual days (*p* ≤ 0.05).

**Table 4:** Individual histologic repair and inflammatory scores of limb wounds following IV allo-CB-MSC administration in horses.

		Histologic Repair Score					Histologic Inflammation Score				
Group	Horse	Day 1	Day 2	Day 7	Day 14	Day 28	Day 1	Day 2	Day 7	Day 14	Day 28
Treatment	2	3	4	8	12	N/A	5	5	4	3	N/A
	3	3	4	9	12	15	5	5	4	3	1
	6	3	4	8	12	15	5	5	4	3	1
	7	3	4	8	11	15	5	5	4	3	1
	8	3	4	8	11	15	5	5	4	3	1
	12	3	4	8	12	15	5	5	4	3	1
Control	1	3	4	8	13	15	5	5	4	1	1
	4	3	4	8	11	15	5	5	4	4	1
	5	3	4	8	11	15	5	5	4	3	1
	9	3	4	8	12	15	5	5	4	3	1
	10	3	4	8	12	15	5	5	4	3	1
	11	3	4	8	11	15	5	5	4	3	1

**Table 5:** GEE analysis of mRNA cytokine expression of limb wounds following IV allo-CB-MSC administration.

	Proinflammatory		Anti-Inflammatory		Inflammation Resolving		Profibrotic		Antifibrotic	
	95% CI	*p* Value	95% CI	*p* Value	95% CI	*p* Value	95% CI	*p* Value	95% CI	*p* Value
Overall Treatment effect	−3.8 to −0.17	0.03 *	−0.33 to −0.025	0.02 *	−0.56 to −0.023	0.03 *	−0.43 to 0.086	0.2 *	−0.72 to 0.29	0.4
Individual Day Treatment Effect	95% CI	*p* Value	95% CI	*p* Value	95% CI	*p* Value	95% CI	*p* Value	95% CI	*p* Value
Day 0	−0.33 to 0.27	>0.9	−0.36 to 0.30	>0.9	−0.67 to 0.51	>0.9	−0.44 to 0.37	>0.9	−0.081 to 0.049	-
Day 1	−10.0 to 9.7	>0.9	−0.94 to 0.36	0.8	−1.4 to 0.48	0.8	−1.3 to 0.78	>0.9	−0.18 to 0.041	-
Day 2	−10.0 to −2.3	<0.001 **	−0.88 to 0.14	0.3	−1.4 to 0.18	0.2	−1.2 to 0.52	0.9	−0.17 to 0.030	-
Day 7	−22 to 10	0.9	−0.64 to 0.12	0.3	−1.1 to 0.15	0.2	−1.1 to 0.32	0.6	−1.4 to 0.46	-
Day 14	−3.3 to 3.7	>0.9	−0.25 to 0.061	0.5	−0.46 to 0.18	0.8	−0.45 to 0.20	0.9	−2.1 to 0.75	-
Day 28	−1.3 to 1.8	>0.9	−0.12 to 0.090	>0.9	−0.47 to 0.47	>0.9	−0.58 to 0.74	>0.9	−2.2 to 2.3	-

(*) = threshold for overall treatment effect over all days (*p* ≤ 0.2). (**) = significant treatment effect on individual days (*p* ≤ 0.05). (-) = data not analyzed because did not meet threshold for overall treatment effect over all days.

## Data Availability

Data used for this study are not publically available, as they are still being used for complementary studies. Data can be made available upon request.
